# Supplementary material for: IFITM proteins inhibit HIV-1 protein synthesis
Source: Sci Rep. 2018 Sep 28;8:14551. doi: 10.1038/s41598-018-32785-5 (PMC6162285; doi:10.1038/s41598-018-32785-5)
Supplement: Supplementary file 1 — Supporting Figures S1-S6 [file 41598_2018_32785_MOESM1_ESM.pdf]

IFITM proteins inhibit HIV-1 protein synthesis

Wing-Yiu Jason Lee, Rebecca Menhua Fu, Chen Liang, Richard D Sloan.

Supporting figures S1-S6

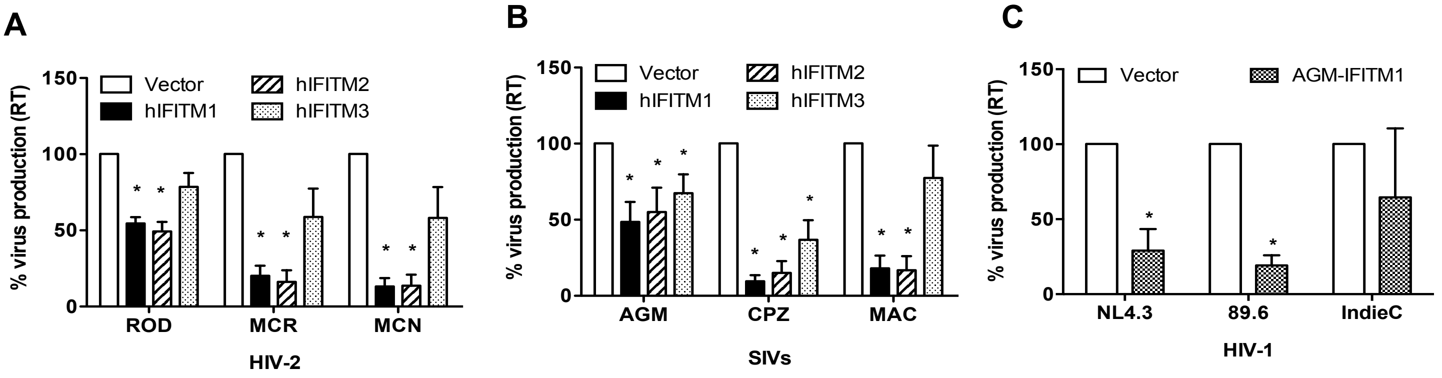

Supporting Fig 1. IFITMs inhibit HIV-1, HIV-2 and SIV viral output. Levels of virus production of HEK293T cells transfected with 0.5μg expression vectors of IFITMs and 0.5μg **(A)** HIV-2 strains or **(B)** SIVs (AGM –African Green Monkey, CPZ – Chimpanzee, MAC – Macaque) proviral DNA were measured by RT activity assay and normalized. **(C)** Levels of virus production of HEK293T cells transfected with 0.5μg expression vectors of African Green Monkey IFITM1 and 0.5μg proviral DNA of HIV-1 were measured by supernatant reverse transcriptase activity assay and normalized. Data shows mean+SEM of 3 independent experiments. Differences were analyzed with Student's t-test and * indicates $p<0.05$.

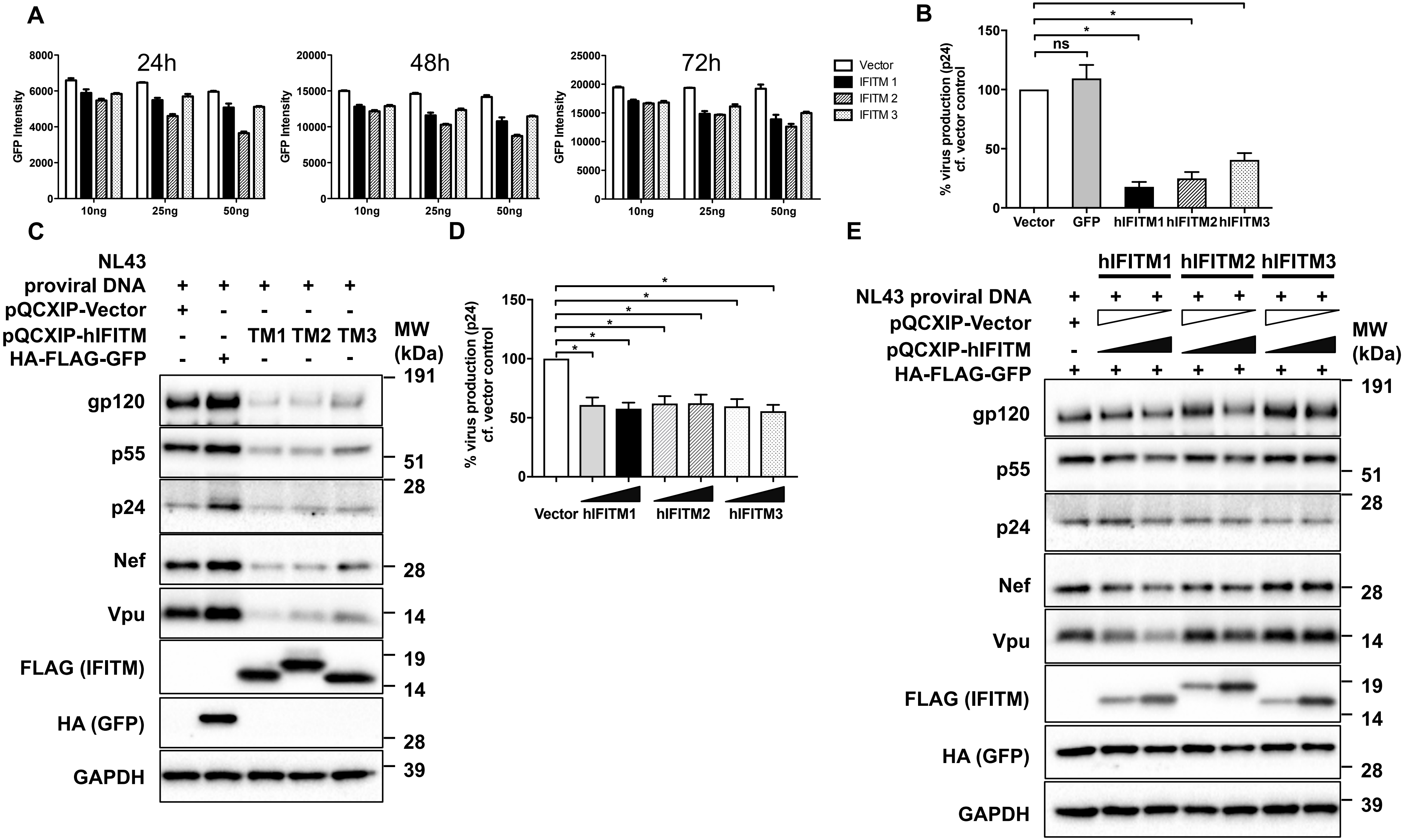
A
24h
48h
72h
GFP Intensity
10ng
25ng
50ng
Vector
IFITM 1
IFITM 2
IFITM 3
B
% virus production (p24) cf. vector control
ns
Vector
GFP
hIFITM1
hIFITM2
hIFITM3
C
NL43 proviral DNA
pQCXIP-Vector
pQCXIP-hIFITM
HA-FLAG-GFP
TM1 TM2 TM3
MW (kDa)
gp120
p55
p24
Nef
Vpu
FLAG (IFITM)
HA (GFP)
GAPDH
D
% virus production (p24) cf. vector control
Vector hIFITM1
hIFITM2
hIFITM3
E
hIFITM1
hIFITM2
hIFITM3
NL43 proviral DNA
pQCXIP-Vector
pQCXIP-hIFITM
HA-FLAG-GFP
MW (kDa)
gp120
p55
p24
Nef
Vpu
FLAG (IFITM)
HA (GFP)
GAPDH

Supporting Fig 2. Expression of human IFITMs does not affect GFP expression in transfected HEK293T cells (A) HEK293T cells were transfected in 96 well plates with 100ng total plasmid DNA per well containing either 10ng, 25ng or 50ng IFITM DNA or vector control (pQCXIP) and 50ng GFP expression vector (pC3-GFP). GFP expression was measured using a plate reader up to 72h post-transfection.

Expression of human IFITMs, but not GFP, in HEK293T cells decreased viral output and viral protein synthesis. HEK293T cells were transfected with 0.5µg of HIV-1 NL4-3 proviral DNA and either 0.5µg of FLAG-tagged IFITM expression vectors or 0.5µg of HA-FLAG-tagged GFP. At 48h post-transfection, levels of virus production in the supernatant were measured by **(B)** p24 ELISA and levels of intracellular viral proteins, IFITMs and GFP were analyzed by **(C)** immunoblotting, GAPDH was used as loading control.

Expression of human IFITMs decreased viral outputs and viral proteins in transfected HEK293T cells without affecting the levels of co-expressed GFP. (D) HEK293T cells were transfected with 0.333µg of HIV-1 NL4-3 proviral DNA, 0.333µg of HA-FLAG-tagged GFP and 0.333µg of FLAG-tagged IFITM expression vectors titrated with the same expression vector without IFITM-encoding sequence. At 48h post-transfection, levels of virus production in the supernatant were measured by **(D)** p24 ELISA; **(E)** levels of intracellular viral proteins, IFITMs and GFP were analyzed by immunoblotting, GAPDH was used as loading control. Data shows mean+SEM of 3 independent experiments. Differences were analyzed with Student's t-test and * indicates $p<0.05$.

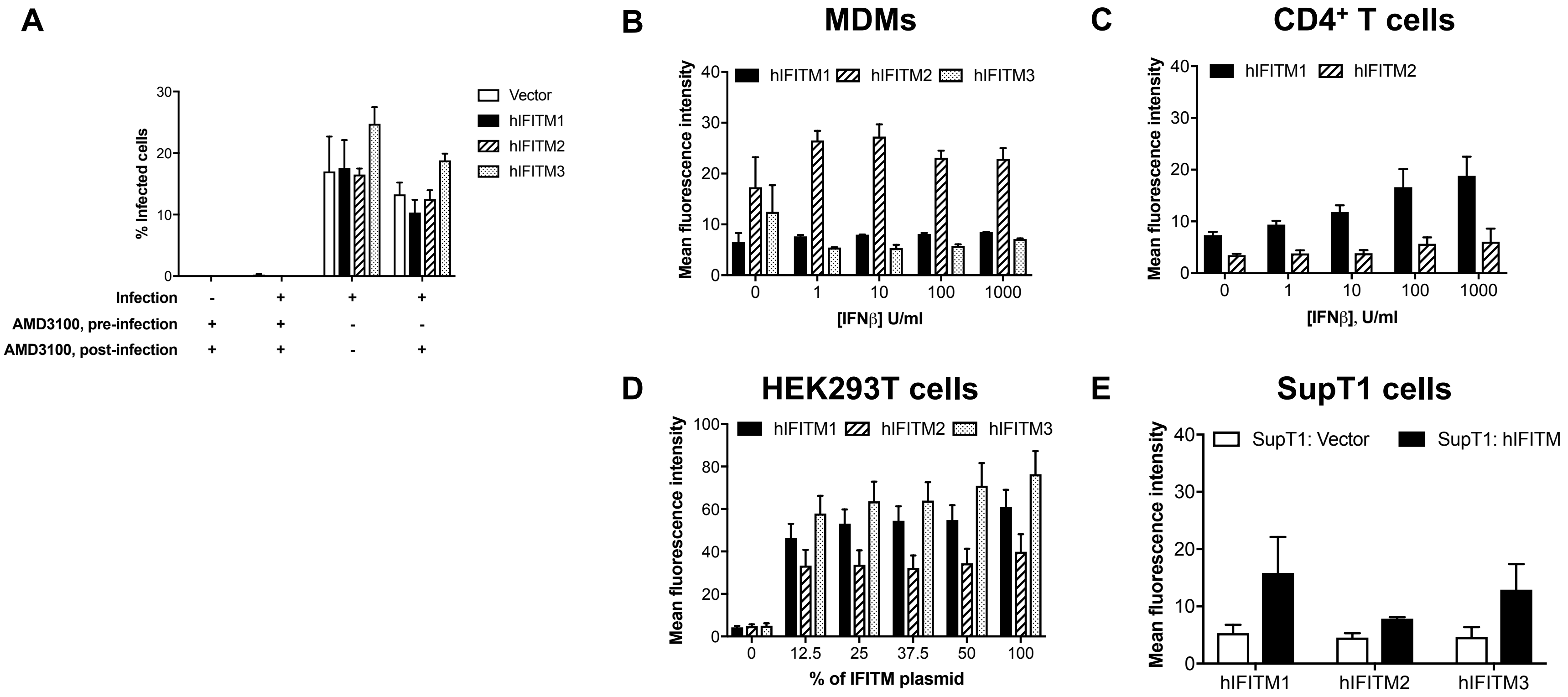

Supporting Fig 3. AMD3100 blocks HIV-1 NL4-3 entry in SupT1 cells. $1x10^6$/ml SupT1 cells were treated with 5µM AMD3100 or DMSO for 2 hours at 37°C and then left untreated or infected with HIV-1 NL4-3 with p24 concentration of 100ng/ml for 2 hours by spinoculation and 1 hour incubation at 37°C. Cells were then washed 3 times with PBS. Levels of virus production were measured by p24 ELISA 48 hours post-transfection. Mean fluorescence intensity of IFITMs in **(B)** human monocyte-derived macrophages (MDMs, day 7) and **(C)** human $CD4^+$ T cells treated with indicated concentrations of IFNβ for 24 hours; **(D)** HEK293T cells transfected with 0.5µg IFITM-encoding plasmids titrated with vector control and 0.5µg HIV-1 proviral DNA for 48 hours and **(E)** SupT1 cells treated with 1µg/ml doxycycline for 48 hours to induce expression of the indicated IFITMs, was measured by intracellular staining of IFITMs with monoclonal antibodies and flow cytometry. Data shows mean+SEM of 3 independent experiments.

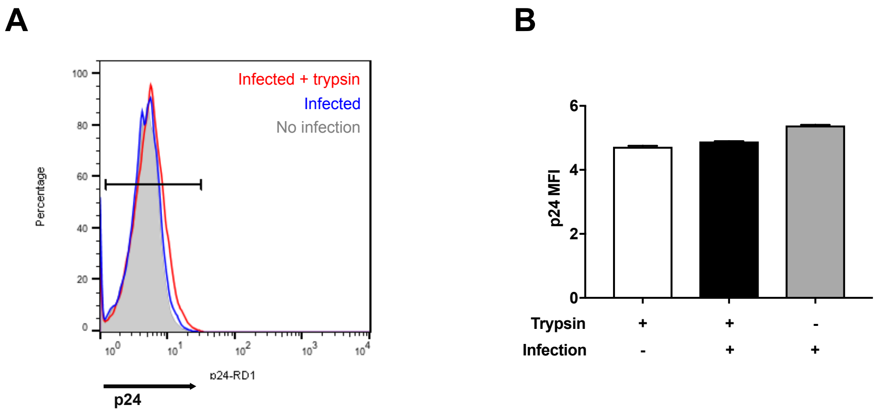

Supporting Fig 4. Trypsin removes surface-bound virus on CD4⁺T cells. 5x10^5/ml human CD4$^+$ T cells were infected or mock infected (medium alone) with NL4-3 wildtype virus at 100ng/ml p24, by spinoculation at 1000 x *g* for 2h at 37°C followed by 1h incubation at 37°C. Cells were then washed with PBS and incubated in 0.25% trypsin, or PBS only for 15 minutes at 37°C and then washed. After fixation with 4% PFA for 10 minutes at room temperature, cells were stained with RD-1-labelled anti-p24 and analyzed by flow cytometry. **(A)** Representative histogram showed overlay of RD-1 fluorescence intensity of infected cells with trypsin treatment (red), infected cells with no trypsin treatment (blue) and non-infected cells (grey). (B) Mean + S.E.M. of p24-RD-1 median fluorescence intensity (MFI) from three independent experiments.

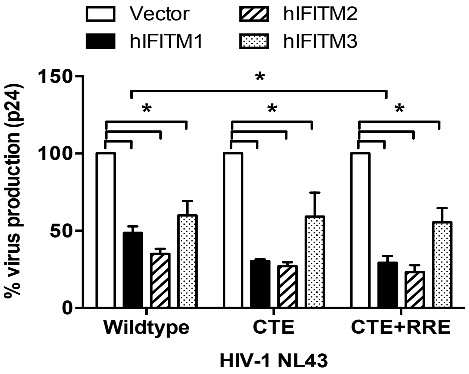

Supporting Fig 5. Substituting the MPMV CTE for the HIV-1 RRE does not affect IFITM-mediated inhibition of HIV-1 production. HEK293T cells were transfected with expression vectors for IFITMs and HIV-1 NL4-3 DNA (wild type [*i.e.* RRE bearing], MPMV CTE only or CTE+RRE). Level of viral production was measured by p24 ELISA 48 hours post-transfection and normalized. Data shows mean+SEM of 3 independent experiments. Differences were analyzed with Student's t-test and * indicates $p<0.05$.

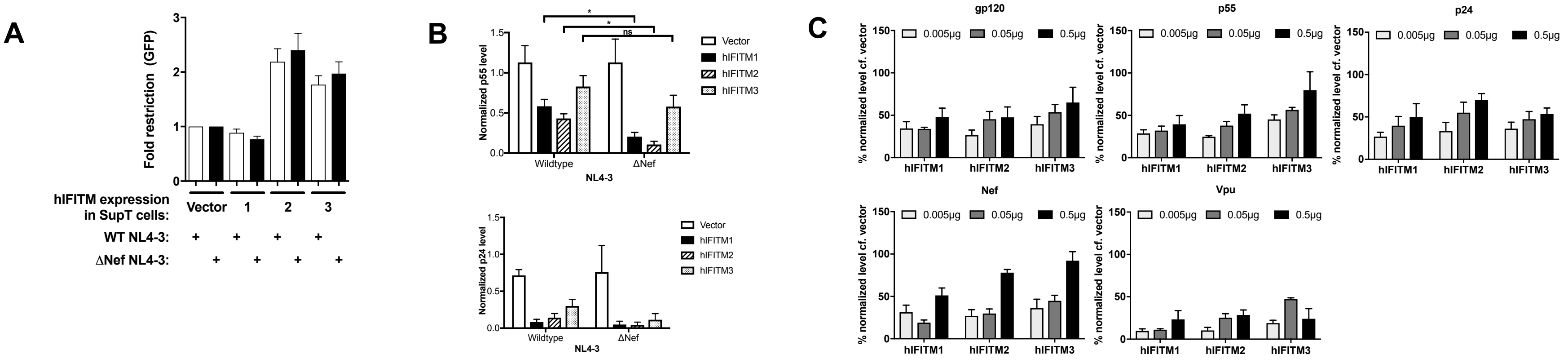

Supporting Fig 6. (A) HIV-1 Nef does not overcome IFITM-mediated inhibition of early viral replication steps. C8166 $CD4^+$ T-cells were transduced to constitutively overexpress IFITM1-3. Cells were then infected with the wild type pBR4-3-eGFP-Nef virus or pBR4-3-eGFP-ΔNef virus. Cells were measured for GFP expression 48h post-infection by FACS to determine infection rate. Data shows fold-change in restriction of GFP infection rates during IFITM expression relative to empty vector control cells. **(B)** Levels of p55 and p24 in immunoblotting of Fig. 7B were quantified by densitometry and normalized to β-actin. Data shows mean + SEM of the ratio of viral proteins to β-actin of 3 independent experiments and differences were assessed by Student's t-test, * denotes $p<0.05$. **(C) Increasing Nef levels rescues HIV-1 protein production during IFITM expression.** HEK293T cells were transfected with ΔNef HIV-1 NL4-3 proviral DNA, expression vectors for IFITMs and an increasing proportion of HIV-1 Nef-encoding vector versus empty vector in a fixed total quantity. Levels of viral proteins and IFITM-FLAG expression in Figure 7G was analyzed by densitometry. Data show mean + SEM of 3 independent experiments and differences were assessed by Student's t-test, * denotes $p<0.05$.
